# Sleep Research Society Statement on Diversity, Equity, and Inclusion

**DOI:** 10.1093/sleepadvances/zpaa003

**Published:** 2020-07-04

Dear *SLEEP Advances* Readers,

We are saddened and outraged at the recent tragedies and deaths due to racialized violence in our country. We are especially horrified by the video of George Floyd’s brutal death. Heartened by ensuing peaceful protests, we were then distressed by outbreaks of violence that followed. These events are a sobering reminder of the challenges that our country must face to achieve racial equity and justice. It is up to each of us, individually and collectively, to address injustices to the best of our abilities.

We affirm our commitment to equity, diversity, and inclusion within our society. The SRS strategic plan specifically names diversity and inclusion as a strategic priority for our society. We recently established a Diversity and Inclusion Task Force to assist the SRS in developing statements, policies, and actions that encourage diversity and inclusiveness in both the membership of our society and the viewpoints we represent.

We pledge to our members that we will continue to promote a respectful and inclusive scientific society, safe from racism and hate. We want all of our members to feel welcome and respected, without barriers to collaboration and the free exchange of ideas. Our goal is to give every researcher—regardless of race, nationality, sexual identity, background, and all other forms of diversity—opportunities and encouragement to be successful sleep and circadian scientists.

Sincerely,

Sleep Research Society Board of Directors

